# Phase I/IIa Feasibility Trial of Autologous Quality- and Quantity-Cultured Peripheral Blood Mononuclear Cell Therapy for Non-Healing Extremity Ulcers

**DOI:** 10.1093/stcltm/szab018

**Published:** 2022-02-26

**Authors:** Rica Tanaka, Satoshi Fujimura, Makiko Kado, Taro Fukuta, Kayo Arita, Rie Hirano-Ito, Tomoya Mita, Hirotaka Watada, Yoshiteru Kato, Katsumi Miyauchi, Hiroshi Mizuno

**Affiliations:** Division of Regenerative Therapy, Juntendo University Graduate School of Medicine, Tokyo, Japan; Department of Plastic and Reconstructive Surgery, Juntendo University School of Medicine, Tokyo, Japan; Intractable Disease Research Center, Juntendo University Graduate School of Medicine, Tokyo, Japan; Division of Regenerative Therapy, Juntendo University Graduate School of Medicine, Tokyo, Japan; Intractable Disease Research Center, Juntendo University Graduate School of Medicine, Tokyo, Japan; Department of Plastic and Reconstructive Surgery, Juntendo University School of Medicine, Tokyo, Japan; Department of Plastic and Reconstructive Surgery, Juntendo University School of Medicine, Tokyo, Japan; Division of Regenerative Therapy, Juntendo University Graduate School of Medicine, Tokyo, Japan; Intractable Disease Research Center, Juntendo University Graduate School of Medicine, Tokyo, Japan; Division of Regenerative Therapy, Juntendo University Graduate School of Medicine, Tokyo, Japan; Center for Genomic and Regenerative Medicine, Juntendo University Graduate School of Medicine, Tokyo, Japan; Department of Metabolism and Endocrinology, Juntendo University Graduate School of Medicine, Tokyo, Japan; Department of Metabolism and Endocrinology, Juntendo University Graduate School of Medicine, Tokyo, Japan; Department of Internal Medicine, Division of Cardiology, Juntendo University School of Medicine, Tokyo, Japan; Department of Internal Medicine, Division of Cardiology, Juntendo University School of Medicine, Tokyo, Japan; Department of Plastic and Reconstructive Surgery, Juntendo University School of Medicine, Tokyo, Japan; Intractable Disease Research Center, Juntendo University Graduate School of Medicine, Tokyo, Japan

**Keywords:** autologous stem cell transplantation, peripheral blood stem cell, vascular development, clinical trial

## Abstract

Non-healing wounds are among the main causes of morbidity and mortality. We recently described a novel, serum-free ex vivo expansion system, the quantity and quality culture system (QQc), which uses peripheral blood mononuclear cells (PBMNCs) for effective and noninvasive regeneration of tissue and vasculature in murine and porcine models. In this prospective clinical study, we investigated the safety and efficacy of QQ-cultured peripheral blood mononuclear cell (MNC-QQ) therapy for chronic non-healing ischemic extremity wounds. Peripheral blood was collected from 9 patients with 10 chronic (>1 month) non-healing wounds (8 males, 1 female; 64-74 years) corresponding to ischemic extremity ulcers. PBMNCs were isolated and cultured using QQc. Within a 20-cm area surrounding the ulcer, 2 × 10^7^ cells were injected under local anesthesia. Wound healing was monitored photometrically every 2 weeks. The primary endpoint was safety, whereas the secondary endpoint was efficacy at 12-week post-injection. All patients remained ambulant, and no deaths, other serious adverse events, or major amputations were observed for 12 weeks after cell transplantation. Six of the 10 cases showed complete wound closure with an average wound closure rate of 73.2% ± 40.1% at 12 weeks. MNC-QQ therapy increased vascular perfusion, skin perfusion pressure, and decreased pain intensity in all patients. These results indicate the feasibility and safety of MNC-QQ therapy in patients with chronic non-healing ischemic extremity wounds. As the therapy involves transplanting highly vasculogenic cells obtained from a small blood sample, it may be an effective and highly vasculogenic strategy for limb salvage.

## Graphical Abstract

**Graphical Abstract:**
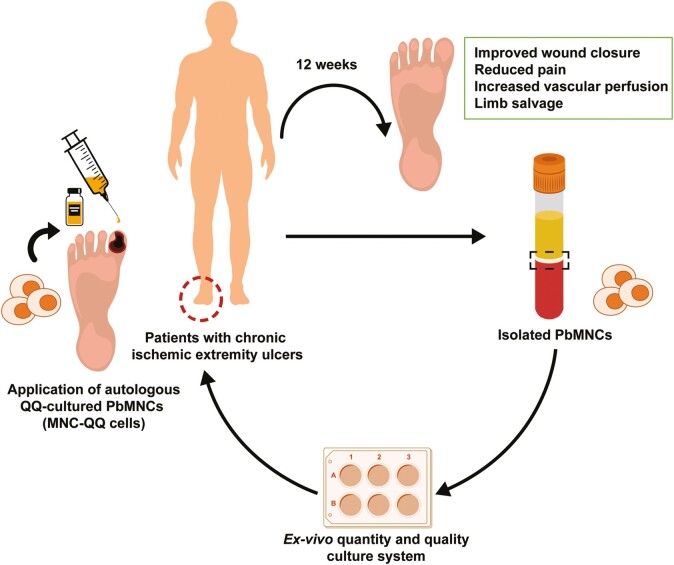
Safety and efficacy of autologous QQ-cultured PBMNC (MNC-QQ therapy) for non-healing ischemic extremity wounds. PBMNCs isolated from patients with ischemic extremity ulcers were cultured in the ex vivo quantity and quality culture system. Autologous PBMNC therapy resulted in improved wound closure, decreased pain intensity, and increased vascular perfusion.

**Significance Statement**

Non-healing extremity wounds impose considerable social and economic burdens. These wounds are difficult to treat and can lead to limb amputation, especially in patients with diabetes. This first-in-human-prospective clinical study provides preliminary but promising evidence for the use of minimally invasive and effective serum-free ex vivo quantity and quality cultured peripheral blood mononuclear cells to treat non-healing chronic extremity wounds in patients with limb ischemia. The outcomes of this trial demonstrate the safety and feasibility of this cell therapy for treating ischemic non-healing extremity wounds.

## Introduction

Treating non-healing extremity wounds related to diabetic ulcers and peripheral vascular diseases is difficult,^1^ and these wounds result in considerable annual economic burdens.^2^ The underlying etiology of diabetic foot ulcers may include peripheral neuropathy and/or peripheral arterial disease (PAD)-associated ischemia. Each of these conditions is related to various factors that affect the ulceration and healing process, highlighting the need for etiological classifications of the patients.^3^ Overall, diabetic ulcers and wound healing can be managed through patient education, treatment of pre-ulcerative symptoms, use of therapeutic footwear or walking aids, and exercise.^4^ However, current treatments also include endovascular treatments, such as percutaneous transluminal angioplasty (PTA) and hyperbaric oxygen therapy^5^ to induce revascularization. Treatment failure leads to gradual tissue necrosis that can require limb amputation.^6^ The 5-year mortality rates post-amputation of lower extremity in diabetes, chronic limb-threatening ischemia (CLTI), and PAD span from 39% to 68%.^7-9^ Thus, effective treatment options for non-healing ischemic ulcers are required.^10^

As the non-healing phenotype of diabetic foot ulcers is related to the lack of vascular regeneration, cell-based therapy involving endothelial progenitor cells (EPCs), an immature vascular stem cell population with the capacity to support angiogenesis by enhancing differentiation into mature endothelial cells or by alternative mechanisms such as paracrine pathways, is considered as a promising treatment approach. EPCs can have multiple progenitor populations, including myeloid cells, hematopoietic progenitor cells, or other tissue-resident EPCs.^11^ Based on their specific origins and distinct epigenetic cues present in the microenvironment, EPCs can be extracted from different sources such as the patient’s own mononuclear cells (MNCs) present in peripheral blood (PB) or bone marrow (BM) using different biomarkers such as CD34^+^ and CD133^+^.^12,13^ Preliminary animal studies used systemic and locally transplanted EPCs to demonstrate the potential of EPC therapy in promoting angiogenesis and wound healing.^14,15^ Clinical trials have explored the use of both of these cell types in promoting vascular and tissue regeneration to treat non-healing diabetic ulcers.^10^ The efficacy and safety of autologous MNCs resulting from BM or PB, and granulocyte colony-stimulating factor (G-CSF)-mobilized PB CD34^+^ cells have been explored in patients with diabetes.^16,17^ However, autologous EPC therapy in patients with diabetes is limited by the relatively weakened mobilization and lower vasculogenic potential of diabetic EPCs compared with healthy EPCs.^18,19^

EPCs comprise only 0.01% of cells in the PB and 0.1% of cells in the BM. Thus, large volumes of BM aspirate, PB apheresis, or injected G-CSF are required to obtain adequate numbers of functional EPCs.^17^ This is particularly difficult in diabetes patients and constitutes a limiting factor in treatment.^20^ Additionally, peripheral blood mononuclear cell (PBMNC) populations are heterogeneous and contain immunogenic cells that can cause inflammatory side effects after cell therapy.^18,19^

To overcome these limitations of EPC dysfunction while treating chronic non-healing extremity wounds in diabetes patients, we established a new method of quality and quantity (QQ) culture to generate EPCs with greater vasculogenic and angiogenic potential.^19,21^ PBMNCs harvested after QQ culture (MNC-QQ) were enriched in CD34^+^, CD133^+^, and CD206^+^ viable cell populations but with fewer colony-forming unit (CFU) numbers of multiple hematopoietic cell populations, including proinflammatory monocytes/macrophages, B lymphocytes, and natural killer cells.^22,23^ We previously demonstrated the efficacy of MNC-QQ cells as a promising therapy for ischemic diseases in large animal studies.^22,24^

This phase I/IIa clinical trial was conducted to investigate the efficacy and safety of autologous MNC-QQ cell therapy in diabetes patients who had chronic non-healing ischemic extremity wounds. To the best of our knowledge, this is the first human trial of a 1-week suspension-based serum-free ex vivo QQ-cultured highly vascularized PBMNC therapy for lower extremity chronic wounds. Therapy can be performed using limited blood aspirations from patients.

## Materials and Methods

### Study Design

This prospective phase I/IIa study was conducted from November 2014 to July 2017 at the Department of Plastic and Reconstructive Surgery, Juntendo University School of Medicine, Tokyo, Japan. The protocol was approved by the appropriate ethics committee and complied with the recommendations of the Declaration of Helsinki. All participants provided written informed consent. The trial was registered with the University Hospital Medical Information Network Clinical Trial Registry (ID: UMIN000016665, March 4, 2015). The study was approved by the Japanese Ministry of Health, Labor, and Welfare under the Human Stem Cell Ethics Guidelines, and the protocol was submitted following the Act on the Safety of Regenerative Medicine.

### Patients

Patients with non-healing chronic ulcers on their extremities were prospectively enrolled in the study. Before enrollment in the study, all patients received appropriate therapy involving clinical care from a cardiologist, plastic surgeon, internal medicine doctor, rehabilitation specialist doctor, and offloading by a prosthetist. The decision process of patient inclusion was performed by a third-party committee of more than 20 members, including doctors (hospital directors, cardiologists, internal medicine doctors, plastic surgeon dermatologists) and nurses. The members of this committee responsible for making the decision were not involved in subsequent cell therapy or in the trial. The inclusion criteria were as follows: age of 20-75 years, strict glycemic control with hemoglobin A1c (<8.0%), and presence of CLTI wounds with Rutherford category 5-6 that were deeper than the subcutaneous layer and were being treated by a wound care specialist following the International Working Group on the Diabetic Foot guidelines for PAD and diabetic foot disease^25,26^ by a wound care specialist for at least 1 month before enrollment, with <60% wound closure. Before cell therapy, all patients underwent PTA, which was approved for only 4 weeks or more before cell therapy. All patients received optimal treatment, and all available treatment modalities were exhausted during treatment. To minimize bias, we waited 4 weeks before assessing the patients to determine whether conventional treatments, including PTA, were effective for closing the wound by >50%. Conventional therapy, except for PTA, was continued during the trial.

Exclusion criteria were malignant diseases, compromised cardiac function (ejection fraction <25% of normal), history of interventional treatment for coronary or cerebral artery stenosis in the last 6 months, diabetic retinal bleeding, hematological disorders, myocardial or cerebral infarction in the last 6 months, complete occlusion of 3 major lower limb arteries, wound infection, severe lung or liver disease, and hemoglobin level of less than 10.0 g/dL. Other exclusion criteria were pregnancy and active infection with hepatitis B or C, human immunodeficiency virus, human T-lymphotropic virus, or parvovirus B19. None of the patients were prescribed any medication changes before or after MNC-QQ therapy. This study did not include a control group, as it was the first-in-human clinical trial to assess safety.

### Study Procedure

Screening was performed by a dedicated group of clinicians. Screening began at 1 month before treatment and included a review of medical history, fulfillment of inclusion and exclusion criteria, and evaluation of vital signs, medications, physical examination, urinalysis, chest x-ray, and hematological and clinical chemistry profiles. Patients underwent cardiac and abdominal echography, fundoscopy, upper gastrointestinal endoscopy, and cerebral, chest, and abdominal computed tomography. During the screening period, patients received standard wound care from a specialist. Minor debridement (or maintenance debridement) of necrotic tissue was performed during regular wound care but major debridement was performed during or after cell transplantation. Because debridement in an ischemic tissue causes enlargement of necrosis, it should be performed when the tissue is well-vascularized. Foot ulcers were photographed, and the area of the ulcer was calculated using a VH analyzer (Keyence Corp., Osaka, Japan).^17^ Vascular perfusion was assessed at baseline and during follow-up by measuring skin perfusion pressure (SPP), ankle-brachial pressure index (ABPI), and transcutaneous oxygen pressure (TcPO_2_).^17^ Angiography of the external iliac artery was performed using an intra-arterial-digital subtraction angiography technique based on the standard Seldinger approach of injecting iohexol (300 mg iodine per milliliter; Omnipaque, Daiichi Sankyo, Japan).

### Preparation of MNC-QQ Cells

Preparation of PBMNCs, QQ culture to produce MNC-QQ cells, and harvesting and collection of cells for lot release tests were performed in a biosafety cabinet or in an isolator system in a controlled hygienic environment at the Cell Processing Center (CPCs) of Juntendo University or Tokyo Medical and Dental University.

PB (100 mL) was collected in EDTA-Na-coated vacuum collection tubes from all participants, twice per day, with a minimum of 4 hours between draws (total 200 mL of PB). The tubes were immediately transported to the CPC for MNC-QQ cell preparation. QQ culture of MNC was performed as described previously.^22^ Briefly, fresh PBMNCs (pre-QQc) were seeded at a density of 2 × 10^6^ cells/well in 6-well Primaria plates (BD Biosciences, Franklin Lakes, NJ, USA) containing 2 mL/well of serum-free Stemline II medium (Sigma-Aldrich, St. Louis, MO, USA) supplemented with recombinant human vascular endothelial growth factor (50 ng/mL), rh Fms-related tyrosine kinase-3 ligand (100 ng/mL), rh interleukin-6 (20 ng/mL), rh thrombopoietin (20 ng/mL), and rh stem cell factor (100 ng/mL) (all from PeproTech, Rocky Hill, NJ, USA), and an antibiotic cocktail (Invitrogen, Carlsbad, CA, USA). The cells were cultured for 7 days at 37°C in a 5% CO_2_ atmosphere.^22^ The cells were harvested and repeatedly washed with EDTA-PBS and saline. After counting, MNC-QQ cells were suspended in saline for injection at a concentration of 2 × 10^7^ cells in 5 mL.

### Quality Analysis of Cells

To ensure the absence of contamination before lot release, PBMNCs from each CPC were free of mycoplasma, as detected using the MycoTOOL PCR Mycoplasma Detection Prep Kit and MycoTOOL PCR Mycoplasma Detection Amp Kit (Roche Diagnostics, Mannheim, Germany), and free of endotoxins as detected using a rapid test (Endosafe nexgen-PTS, Charles River Laboratories, Wilmington, MA, USA). Endotoxins were also tested using the Endospecy ES-50M set (Seikagaku Corp., Tokyo, Japan) as an outsourcing test at the SRL facility in Hachioji, Japan. All tests were performed according to the manufacturer’s protocol. For the sterility test, 10 mL of the supernatant from the final centrifugation process was collected in BACTEC plus aerobic or anaerobic culture medium bottles (BD Biosciences) and cultured for 14 days.

### Product Tests for MNC-QQ

Release tests for MNC-QQ were conducted to evaluate potency based on the ratio of cells and their vasculogenic potential. The presence of cell types essential for vascular and tissue regeneration in the MNC-QQ culture was confirmed using flow cytometry antibody-based detection of the following cell surface markers: CD34-PE, CD206-PE/Cy7, CD192(CCR2)-PerCP/Cy5.5, CD3-Alexa700, CD14-APC/Cy7, CD4-FITC, CD8-APC/Cy7, CD31-APC/Cy7, CD19-FITC, CD56-BV421, CD25-PerCP/Cy5.5, CD127-BV421 (BioLegend, San Diego, CA, USA), CD133-APC (Miltenyi Biotec, San Diego, CA, USA), and CXCR4-APC (BD Biosciences).^17,23^

The vasculogenic potential of MNC-QQ was evaluated using a MethoCult SF H4236 colony formation assay (STEMCELL Technologies, Vancouver, BC, Canada).^22^ Cells (2 × 10^5^ per dish) were inoculated into a 35-mm Primaria culture dish and cultured for 14 days under routine conditions. Clusters comprising more than 100 cells were counted as primitive EPC CFU or definitive CFU based on the cell size and shape under phase-contrast microscopy (Nikon, Tokyo, Japan). PB collected less than 1 month before cell therapy was used to compare the baseline vasculogenic potential and cell surface markers between PBMNCs and MNC-QQ cells harvested on the treatment date.

### Treatment

After debridement and irrigation of the wound(s), patients underwent MNC-QQ transplantation under general anesthesia on the same day as cell harvest from the CPC. MNC-QQ cells were intramuscularly injected within a circumference of 20 cm around the wound using a 27-gauge needle. Each patient was administered a total of 2 × 10^7^ MNC-QQ cells via injections at 20 sites (1.5-2.0 cm deep). Each injection contained 1 × 10^6^ cells suspended in 0.25 mL saline. In case of limited availability of cells, the cell concentration was reduced to 1 × 10^5^ cells in 0.25 mL saline. A saline gauze was immediately placed over the treated wound to prevent cell damage. Standard wound care was continued from day 1 onward, post-treatment. Patients were discharged from the hospital the following day, provided there were no side effects from the MNC-QQ cell therapy. The standard care regimen for diabetic feet continued from the day of discharge.

### Endpoints and Follow-up

The primary endpoint was the treatment safety. The secondary (evaluation) endpoint was treatment efficacy at 12 weeks after therapy. This evaluation time point was determined based on published clinical trials on diabetic wound healing.^27,28^ Safety was evaluated based on the incidence of adverse events (AEs), and the severity of AEs was evaluated according to the National Cancer Institute Common Terminology Criteria for Adverse Events (NCI-CTCAE, version 3).

The pre-specified efficacy score was a surrogate endpoint that used both objective and subjective parameters. The efficacy score was calculated as the sum of 6 sub-scores. Each of the sub-scores was measured as the difference in the following parameters between baseline and its respective end point after cell therapy: (1) percent wound closure after 12 weeks,^29^ (2) SPP in the treated foot after 12 weeks, (3) Wong-Baker FACES Pain Rating Scale score for pain assessment in the treated leg^9^ after 12 weeks; (4) recurrence of the treated wound within 1 year or more; (5) amputation-free survival at 1 year after therapy; and (6) recurrence of ischemia within 1 year or more. Two physicians, who were not part of the study, validated the efficacy score (Supplementary Table 1).

### Evaluation of Parameters

Treated patients visited the clinic at 2-, 4-, 8-, and 12-week post-injection for safety and efficacy evaluations and to assess wound recurrence. Percent wound closure, ABPI, SPP, and TcPO_2_ were measured at each visit. Angiographic analysis was performed at 12 weeks in patients with severe peripheral vascular disease. Pain level was evaluated at baseline and 12 weeks after transplantation. A clinical research coordinator interviewed each patient to obtain information on psychoesthesia, paresthesia, and analgesic drug requirements at baseline and 12 weeks after transplantation. In the absence of wound closure at 12 weeks, follow-up was continued once every 4 weeks until complete closure. Recurrent wounds received standard wound care, and the time to wound closure was evaluated. The total follow-up period was more than 1 year.

### Statistical Analyses

All data are presented as the mean ± standard deviation. Student *t* test was performed to assess statistically significant differences in cell marker expression between the 2 groups of cells (PBMNC and MNC-QQ). The paired-sample *t* test was used to compare the measured numerical parameters between the baseline and follow-up. One-way analysis of variance for repeated measurements was used when comparisons involved more than 2 groups. The correlation between the number of cells expressing cell markers of interest and efficacy score was analyzed using correlation analysis. Amputation-free survival and cardiovascular event-free survival were calculated using the Kaplan-Meier method. Statistical significance was defined as *P* < .05. Graph Pad Prism, ver. 5 (GraphPad Software, Inc., La Jolla, CA, USA) was used for all statistical analyses.

## Results

### Patient Characteristics

Tables 1 and 2 show the characteristics of the 9 patients (corresponding to ulcers in 10 limbs) enrolled in the trial. Eight patients presented with CLTI (ulcers in 9 limbs), 7 had diabetes, 8 required hemodialysis for chronic renal failure, and 2 had scleroderma. Wounds were predominantly located on toes, except in cases 4 (heel) and 8 (finger). The blood sugar levels of all patients with diabetes were adequately controlled, with hemoglobin A1c levels less than 8.0%. The average wound size was 3.0 ± 2.1 cm^2^ and the average wound duration before MNC-QQ cell therapy was 225.6 ± 265 days. The average ABPI was 0.70 ± 0.1. All patients exhibited low SPP (26.6 ± 10.9 mmHg), and TcPO_2_ values proximal to the wound (30.4 ± 11.8 mmHg), indicating wound ischemia. All cases, except for cases 5 and 9, had PTA for greater than 1 month before therapy.

**Table 1.** Baseline characteristics of patients.

Characteristic	*N* (number of cases) = 10
Age (yr)	60 ± 7
Male/female	9/1
CLTI wounds	9
Rutherford 5	6
Rutherford 6	3
Upper limb wound	1
Collagen disease (scleroderma)	2
Hypothyroidism	2
Catheter intervention	9
Bypass surgery	0
Chronic renal failure on hemodialysis	8
Hypertension	4
Diabetes mellitus	7
HbA1c%	6.2 ± 1.0
Hyperlipidemia	3
Smoking	0
Coronary artery disease	7
CABG surgery	5
EF%	55.7 ± 16
AF	1
Cerebral artery disease	4
Clopidogrel sulfate (ADP inhibitor)	3
Ethyl icosapentate (EPA)	3
Cilostazol	3
Aspirin	7
Limaprost alfadex (PGE1 derivative, prostanoid)	1
Atorvastatin (statin)	2
Pitavastatin (statin)	1
Warfarin	5
Carvedilol (αβ-blocker)	3
Sarpogrelate (5-HT2 antagonist)	1
Nifedipine (Ca channel blocker)	1

Note: Total number of patients = 9; *N* (number of cases) = 10; cases 2 and 4 correspond to the same patient.

Abbreviations: AF, atrial fibrillation; CABG, coronary artery bypass grafting; CLTI, chronic limb-threatening ischemia; EF, ejection fraction; EPA, eicosapentaenoic acid; PGE1, prostaglandin E1.

**Table 2.** Clinical history and outcomes in all patients treated with autologous peripheral blood MNC-QQ cell therapy.

	Pre-therapy										Post-therapy							
Cases	Sex	Age (yr)	Location of wound	Wound size (cm^2^)	Major medical history	HD	EF	Number of interventions before MNC-QQ therapy	Area of stenosis (% of occlusion)	Wound history before MNC-QQ therapy (days)	% Wound closure post 3M	Pain	Vascular perfusion	Improvement in angiogram	Ambulant	Time to complete wound closure	Recurrence within 1 yr	Restenosis within 1 yr
1	M	74	First toe	2.7	DM CLTI	+	73	1	Lt SFA 0% ATA 25% CIA 50% EIA 50% PTA 90%	111	100%	Disappeared	Improved	+	+	110	–	–
2	M	65	First toe	0.4	DM, CLTI	+	57	3	Rt SFA 0% ATA total 20% PTA 100%	221	55%	Improved	Improved	−	+	Deceased without wound closure	–	+
3	M	64	First, second, third, fifth toe	1.1	DM, CLTI	+	35	2	Rt ATA 0% PA 25% PTA 100%	81	−169%	Improved	Improved	−	+	224	–	+
4	M	65	Heel	3.4	DM, CLTI	+	66	3	Lt SFA 25% POA 25% TPT 25% ATA 100% PTA 100%	50	−17%	Improved	Improved	−	+	Deceased without wound closure	–	+
5	M	66	Third toe	0.5	DM, CLTI	+	36	0	N/A	151	100	Disappeared	Improved	NA	+	29	+	–
6	M	64	Fifth toe	3	DM, CLTI	+	53	1	Lt ATA 25% PTA 25% PA 90%	183	100	Disappeared	Improved	−	+	47	+	–
7	F	66	First, second toe fifth metatarsal	5.2	PSS, CLTI	−	77	1	ATA total PTA 100% PA 25%	114	100	Improved	Improved	+	+	63	+	+
8	M	61	First toe	7	DM, CLTI	+	56	2	Lt ATA 0% PTA 25% PA 90%	183	100	Improved	Improved	−	+	26	–	–
9	M	47	Second, third finger	4	PSS	−	74	0	N/A	965	100	Improved	Improved	NA	+	55	–	–
10	M	61	Second, third, fourth, fifth metatarsal post-amputation	2.3	CLTI	+	45	3	Rt ATA total 25% SFA 75% PTA 100%	197	87.4%	Improved	Improved	−	+	179	–	+

Abbreviations: ATA, anterior tibial artery; CHF, chronic heart failure; CIA, common iliac artery; CLTI, chronic limb-threatening ischemia; CRF on HD, hemodialysis; DM, diabetes; EF, ejection fraction; EIA, external iliac artery; Lt, left; NA, not applicable; PA, popliteal artery; POA, popliteal artery; PSS, scleroderma; PTA, posterior tibial artery; Rt, right; SFA, superficial federal artery; TPT, tibial peroneal trunk.

### Product Outcome and Results of Release Tests of MNC-QQ Cells

The 200 mL of PB obtained from each patient yielded an average of 206 ± 51 × 10^6^ MNCs with a viability of 93% ± 9.8%. MNC culture for 1 week under QQc resulted in 36.29 ± 20.04 × 10^6^ harvestable cells, representing a 0.17 ± 0.07-fold increase. The number of isolated MNC-QQ cells was lower than the number in the protocol (2 × 10^7^ cells) in cases 8, 9, and 10. In these 3 cases, all cells available after quality control testing were injected. Cell cultures for all cases tested negative for endotoxin, mycoplasma, and bacteria (Table 2). Fluorescence-activated cell sorting (FACS) data (average ± SD: CD34 = 2.0% ± 1.2%, CD206 19.7% ± 15.6%) indicated that all cultures met the product release test criteria (Supplementary Table 2).

### Vasculogenic Potential and Characteristics of MNC-QQ Cells

**Figure 1.**
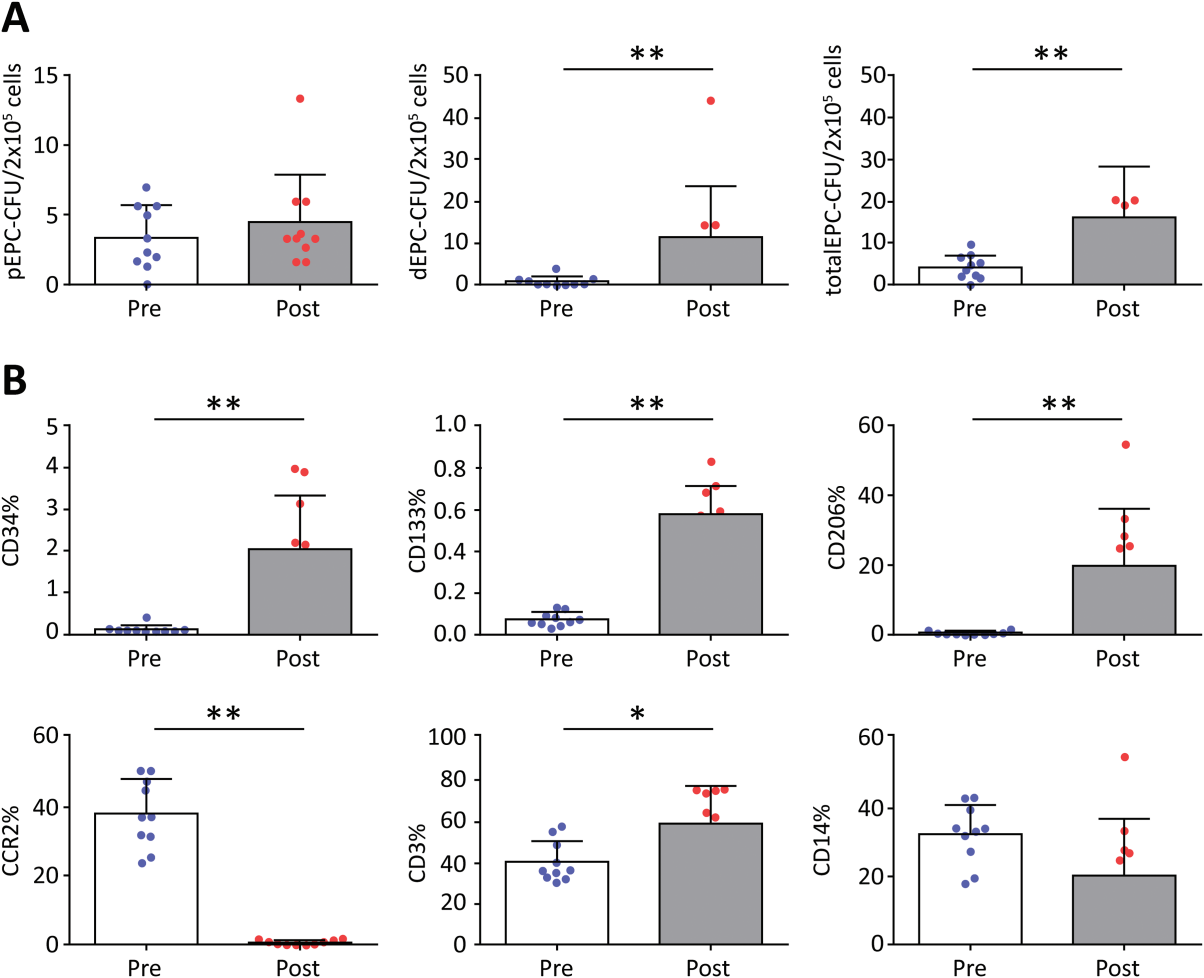
Vasculogenic potential and expression characteristics of MNC-QQ cells. (A) Vasculogenic colony formation assay of PBMNCs collected 1 month before MNC-QQ cell therapy (pre-QQ) and compared with that of MNC-QQ cells (post-QQ). Note that the vasculogenic potential of MNC-QQ cells was significantly higher than that of PBMNCs. (B) FACS analysis of PBMNC (pre-QQ) and MNC-QQ (post-QQ) cells showing the percentage of cells expressing CD34, CD133, CD 206, CCR2, CD3, and CD14 surface markers. Cells from the pre-QQc were collected 1 month before the study, whereas post-QQc treatment data were obtained on the day of cell therapy. Most markers showed greater expression after the QQ protocol. Data are presented as mean ± SD; CD34^+^, CD 133^+^, CD206^+^ (∗∗*P* = .002); CCR2^+^ (∗∗*P* = .002); CD3^+^ (∗*P* = .0195). Abbreviations: MNC, mononuclear cells; PBMNCs, peripheral blood mononuclear cells; QQc, quality and quantity culture.

All cells were cultured, and hence they were collected and transplanted on the same day (the treatment date). The vasculogenic potential of PBMNCs was significantly lower than that of MNC-QQ cells. PBMNCs produced significantly fewer dECU-CFUs (0.89 ± 0.37 vs 11.67 ± 3.80; *P* = .0039) and significantly fewer total CFU-EPCs (4.30 ± 0.93 vs 16.17 ± 3.89; *P* = .039) (Fig. 1A). FACS analysis revealed a significant increase in the following markers of MNC-QQ cells: CD34 (0.11 ± 0.03 vs 2.039 ± 0.405; *P* = .002), CD133 (0.07 ± 0.01 vs 0.576 ± 0.04; *P* = .002), CD206 (0.47 ± 0.13 vs 19.74 ± 5.20; *P* = .002), and CD3 (40.32 ± 3.11 vs 59.17 ± 5.67; *P* = .0195). CCR2 showed a significant increase (38.23 ± 3.17 vs 0.71 ± 0.22; *P* = .002) (Fig. 1B; Supplementary Fig. 1).

### Safety Evaluation

No deaths (NCI-CTCAE grade 5) occurred, and no life-threatening AEs (grade 4) were observed during the 12-week follow-up period after cell therapy (Table 3). Mild to severe AEs (grades 1-3) that were unrelated to the treatment were observed in all patients (Table 3). Grade 3 symptoms included cellulitis at the injection site and development of a wound in the left first toe (case 1). The wound healed completely by day 173 after the therapy. Other symptoms included restenosis in an area previously treated with percutaneous balloon angioplasty and chronic subdural hematoma unrelated to the therapy. All grade 1 symptoms were transient and related to the medical history of the patient rather than to MNC-QQ cell injection. Importantly, all symptoms disappeared over time without permanent damage. No AEs were observed after local anesthesia, and there was no incidence of pathological retinal angiogenesis.

**Table 3.** Adverse events during 12 weeks of follow-up after PB MNC-QQ cell transplantation.

NCI-CTCAE (version 3.0) grade	Adverse events
Grade 5 (death)	None
Grade 4 (life-threatening)	None
Grade 3 (severe)	Cellulitis at an injection site^a^ (*N* = 1) Restenosis (*N* = 4) Chronic subdural hematoma (*N* = 1)
Grade 1-2 (mild to moderate)	*Clinical symptoms* Bedsore (*N* = 1) Heterotopic ulcer (*N* = 1) Urinary tract infection (*N* = 1) Arthralgia (*N* = 1) Dyspnea (*N* = 1) Hypoperfusion (*N* = 1) Labial herpes simplex (*N* = 1) Diarrhea (*N* = 1) Patellofemoral joint pain (*N* = 1) Fever due to respiratory infection (*N* = 1)
	*Laboratory data abnormalities* ALP elevation (hepatic-cystic system failure) (*N* = 1) CRP elevation (*N* = 1)

Note:

Adverse event related to MNC-QQ cell transplantation.

Abbreviations: ALP, alkaline phosphatase; CRP, C-reactive protein; NCI-CTCAE, National Cancer Center Common Terminology Criteria for Adverse Events.

### Efficacy Evaluations

#### Wound Closure

**Figure 2.**
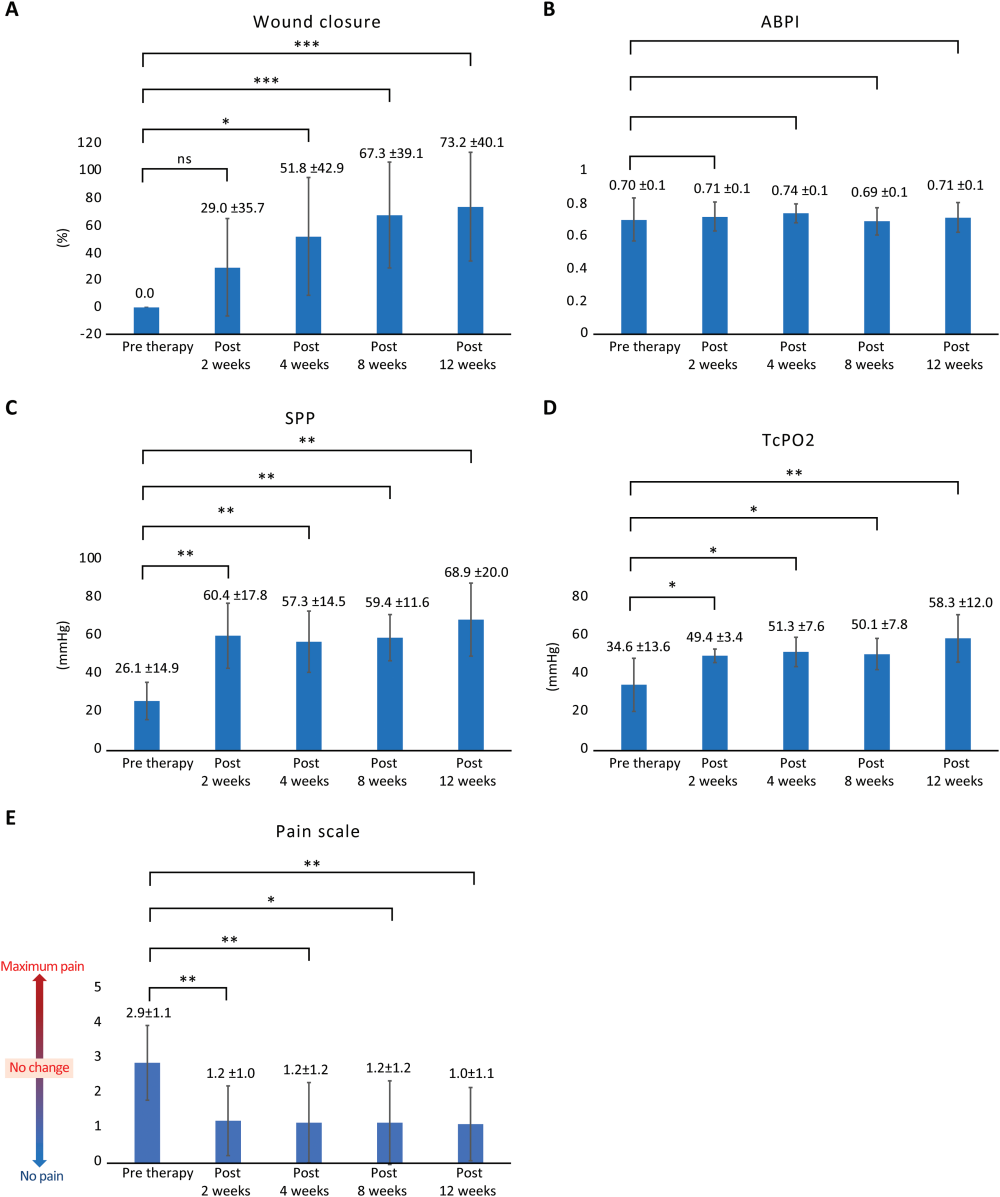
Efficacy of MNC-QQ treatment. The following were evaluated as objective parameters of wound healing. (A) Wound closure at various time points during the 12-week follow-up. ns, not significant; ∗*P* < .05; ∗∗∗*P* < .001. (B) ABPI at various time points during the 12-week follow-up. Case 9 was excluded from this assessment. (C) SPP at various time points during the 12-week follow-up; ∗∗*P* < .01. (D) TcPO_2_ at various time points during the 12-week follow-up; ∗∗*P* < .01. Data are presented as mean ± SD. (E) Pain level was evaluated using the Wong-Baker FACES Pain Rating Scale before and during 12 weeks of follow-up. Pain scale at various time points during the 12-week follow-up. All patients who experienced pain before transplantation reported a significant decrease in pain levels after therapy with PBMNC-QQ cells. Data are presented as mean ± SD (∗∗*P* < .001). Abbreviations: ABPI, ankle-brachial pressure index; MNC, mononuclear cells; PBMNCs, peripheral blood mononuclear cells; QQ, quality and quantity culture; SPP, skin perfusion pressure; TcPO_2_, transcutaneous oxygen pressure.

Complete wound closure occurred in 7 of 10 patients at 12 weeks, at which time the average wound closure rate was 73.2% ± 40.1% (Fig. 2A). Wounds in 9 cases completely closed after an average duration of 91.6 days. The length of time was substantially less than the 248.1 days of non-healing time before cell therapy in these patients. Subjects who had developed gangrene with dry necrosis in the toes underwent minor amputation to completely close the wound. In case 2, restenosis of the right superficial femoral artery (90% occlusion) and posterior and anterior tibial arteries (100% occlusion of both) occurred at 2 months after cell therapy, and the patient underwent PTA. Simultaneously, case 2 also presented with an ischemic ulcer in the left heel due to 100% occlusion of the posterior tibial artery, which did not heal with PTA. Nevertheless, the patient was included in the study as case 4, with an ulcer on the left foot. As indicated by the data in Table 2, patients with a history of multiple PTA procedures before cell therapy tended to develop restenosis of the treated artery, which caused slower wound healing or worsening of the wound despite cell therapy.

#### Peripheral Vascular Perfusion

Although there was no significant change in resting ABPI before and following cell therapy (0.71 ± 0.1 vs 0.71 ± 0.1; Fig. 2B), SPP and TcPO_2_ (proximal to the wound) were significantly higher at 12 weeks compared with the values at baseline. SPP was 30.2 ± 14.9 and 69.9 ± 20.0 mmHg at baseline and post-therapy, respectively (*P* = .0039; Fig. 2C). TcPO_2_ was 34.6 ± 13.6 and 58.3 ± 12.0 mmHg at baseline and post-therapy, respectively; *P* = .0039; Fig. 2D). However, a significant increase in vascularity on angiography was not observed in most cases, except in cases 1 and 7.

#### Pain Scale

All patients complained of limb and foot pain before therapy. Pain began to decrease at 2 weeks after therapy. The average pain level evaluated using the Wong-Baker FACES Pain Rating Scale was 2.9 ± 1.1 at baseline, which decreased to 1.0 ± 1.1 at 12-week post-therapy (*P* < .0078; Fig. 2E).

#### Efficacy Score

**Figure 3.**
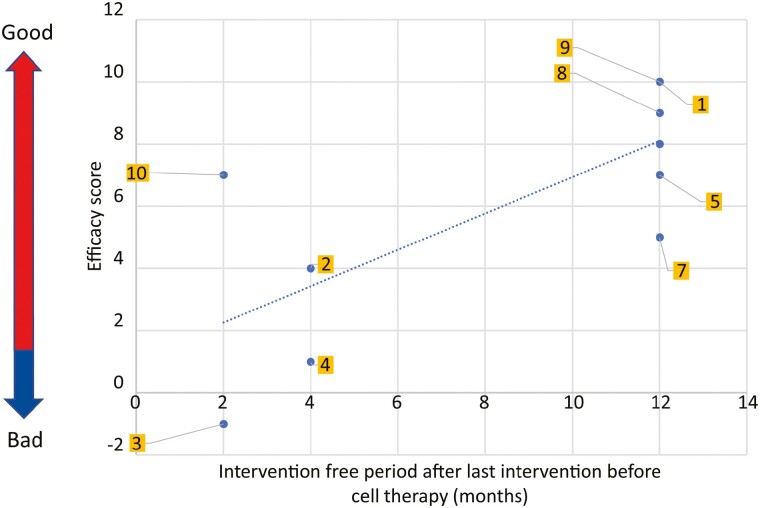
Efficacy evaluation of PBMNC-QQc therapy in patients. Relationship between the intervention-free period after the last intervention before cell therapy and efficacy scores. Individual cases are depicted as blue dots, and the yellow squares denote case numbers. Patients with higher efficacy scores achieved a longer intervention-free period after cell therapy. Abbreviations: PBMNC, peripheral blood mononuclear cells; QQc, quality and quantity culture.

Supplementary Table 3 lists the efficacy scores for all cases. All patients showed improvements in vascular perfusion and pain levels. Patients with higher efficacy had a longer intervention-free period after cell therapy. Lower efficacy scores were evident in patients with severe CLTI who presented with chronic non-healing wounds and developed recurrent restenosis in the lower limb arteries (Fig. 3).

**Figure 4.**
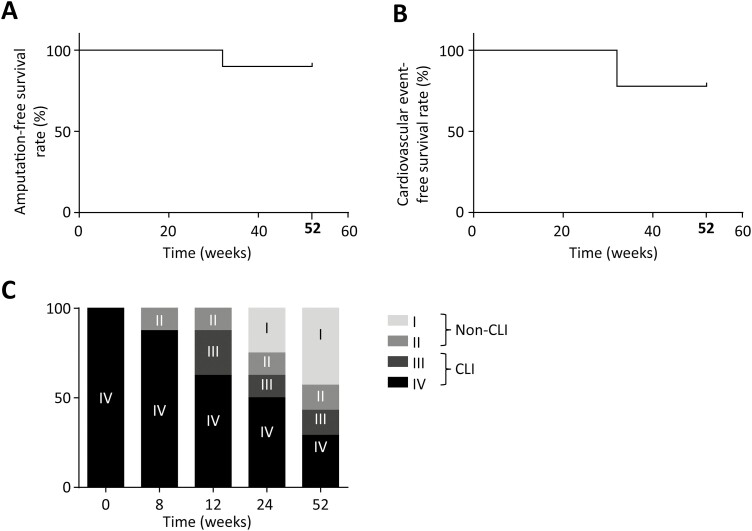
Amputation-free survival, cardiovascular event-free survival, and CLTI-free rate. (A) Amputation-free survival at 1 year was 89%. (B) Cardiovascular event-free survival rate was 78% at 32 and 52 weeks. (C) Fontaine stage and CLTI-free rate. Gray bar indicates CLTI and open bars indicate non-CLTI. The improvement in the CLTI stage to non-CLTI stage at 52 weeks was 57% (4 out of 7 patients). Abbreviation: CLTI, critical limb ischemia.

Supplementary Figure 1 shows the total number of CFUs after QQ treatment in the MNC-QQ cells. No correlation between efficacy score and CFU of MNC-QQ cells (indicating vasculogenic potential) was evident. Moreover, the numbers of CD3-, CD34-, CD133-, CD206-, CD14-, and CCR2-positive cells were not significantly correlated with the efficacy score (Supplementary Fig. 1). Eight (89%) patients survived for 1 year without major or minor amputations (Fig. 4A). The rate of cardiovascular event-free survival was 78% at 32 and 52 weeks (Fig. 4B). The rate of improvement at the CLTI stage to the non-CLTI stage at 52 weeks after cell transplantation was 57% (4/7 patients; Fig. 4C).

## Discussion

Wound healing includes a synchronized interplay of various cellular and biochemical events involving diverse cell types and physiological factors. In an early study of cell-based therapy for wound healing, Holzinger et al showed that topically applied activated autologous PBMNCs facilitated the epithelialization of dermal ulcers.^30^ Our previous study indicated that increased secretion of proangiogenic cytokines for autocrine and paracrine actions enhanced the vasculogenic potential and performance of post-QQc cells and contributed to effective wound healing.^22^ Earlier, in compassionate use of cell-based therapy, topical application of PBMNCs along with basic fibroblast growth factor for treating diabetic gangrene or application of lethally γ-irradiated non-human leukocyte antigen-matched MNCs derived from cord blood in a no-option CLTI patient helped in wound healing.^31,32^ This study was developed following the regeneration therapy law, the Act on the Safety of Regenerative Medicine (Safety Act) of Japan,^33^ and was the first clinical trial of autologous transplantation of MNC-QQ cells with vasculogenic potential in patients with chronic non-healing wounds. The therapy used only 200 mL of the patient’s own blood as the source of MNCs. Although PBMNCs that include diverse multipotent progenitor cells are promising candidates for cell therapy, the physiological condition of the donor severely affects treatment efficiency. Thus, the special treatment of PBMNCs is required before transplantation to ensure that they can promote tissue regeneration and restore function after injury.^34^ The efficacy of MNC-QQ cells in promoting wound healing in large animals suggests their potential for use in humans.^24^ In previous human trials, transplanted autologous MNCs included populations of CD34^+^ or CD133^+^ cells as EPCs.^16,35^ Although therapy with G-CSF-mobilized CD34^+^ cells was efficacious for treating CLTI and diabetic non-healing wounds,^17,36^ the low yield and limited vasculogenic potential of these cells led to an insufficient clinical benefit, particularly in patients with diabetes. We hypothesized that autologous MNCs treated with a serum-free QQ expansion culture would be effective for treating ischemic chronic non-healing wounds. The results of the small, prospective, open phase I/IIa clinical trial showed significant improvement in various relevant parameters after MNC-QQ cell transplantation.

PAD belongs to the pathological spectrum of atherosclerosis and predominantly manifests in the arteries of the lower extremities, leading to CLTI development in at least 10% of the affected population.^37^ Most patients in our study had diabetes or required hemodialysis. SPP and TcPO_2_ were measured as reliable indicators of peripheral vascular perfusion.^38,39^ A lack of change in ABPI after therapy is consistent with a prior report of no relationship between ABPI and severity of peripheral ischemia if the vessels involved were calcified in patients with diabetes or in those undergoing hemodialysis.^17,40^

Although ABPI showed no significant change, SPP and TcPO_2_ increased significantly after MNC-QQ cell therapy. The effects were evident at 2 weeks and persisted for 12 weeks. Furthermore, although patients with peripheral vascular disease and end-stage renal failure are typically less responsive to peripheral or BM MNC therapy,^41^ 60% of patients undergoing hemodialysis in our study showed increased vascular perfusion, reduced pain, and complete wound closure by 12-week post-therapy. Similarly, although patients with CLTI undergoing dialysis or those with unhealed wounds reportedly have a significantly poorer 1-year survival rate of 48%,^9^ the higher 1-year survival rate in our study (8 of 9 patients survived) indicates the clinical utility of MNC-QQ therapy in these patients.

EPC mobilization is mediated by vascular endothelial growth factor and other cytokines released from injured vascular tissue, G-CSF, stromal cell-derived factor-1, and erythropoietin.^42^ Thus, the impaired mobilization and lower vasculogenic potential of diabetic EPCs limit the efficiency of autologous EPC therapy in patients with diabetes.^43-45^ Additionally, in such patients, impaired migration of EPCs leads to diabetic microangiopathy, causing a loss of adhesive intercellular contacts and increased endothelial permeability.^46^ The BM of diabetes patients with CLTI reportedly has fewer CD34^+^ progenitor cells but increased fat deposition.^47^ Whether application of cell-free secretome of apoptotic PBMNCs, which contained various lipids, proteins, cytokines, exosomes, and vasoactive substances, at the wound sites improves healing remains unclear.^48,49^ Interestingly, when studied in experimental acute myocardial infarction, a cell suspension of irradiated apoptotic PBMNCs AMI overcame inflammation, facilitated regenerative EPC homing, and replaced infarcted myocardium.^50^ Nevertheless, our QQ expansion culture system improved the vasculogenic and wound healing properties of these EPCs to levels observed in healthy cells.^19^

The practicality of procedures, such as mesenchymal stem cell therapy, BM MNC or PBMNC therapy, and CD34^+^ cell therapy for treating ischemic diseases remain limited.^51,52^ These treatments require multiple days of injection and apheresis sessions or liposuction and are associated with relatively higher levels of complications, risk, technical challenges, and patient burden. Although MNC or CD34^+^ cell therapy does not include cell culture processes when transplanted, the vasculogenic function of these cells has been estimated to be lower than that of MNC-QQ cells.^17^ Our recent study revealed the clinical applicability of QQc for PBMNCs in patients with diabetes.^23^ Our therapy has some advantages over traditional methods of cell harvesting, particularly for treating patients with non-healing ischemic ulcers. These advantages include the ability to perform the procedure in an outpatient setting and need to draw a total of only 200 mL of blood in two 100-mL volumes from the patients. Importantly, the resultant MNC-QQ cells possess substantial vasculogenic properties.^22^

Although the efficacy of CD34^+^ cell therapy is thought to be correlated with the vasculogenic function of transplanted cells, we found no direct correlation between the efficacy scores and number of EPC-CFUs. Comparison of the number of EPC-CFUs before and after QQc culture showed a significant increase in the number of definitive EPC-CFUs and total EPC-CFUs in MNC-QQ cells of all patients. Although the vasculogenic potential of MNC-QQ cells in all cases reached the threshold required to achieve an increase in vascular perfusion and pain control, certain patient-specific factors affected their efficacy. For example, patients who had undergone more interventions before cell therapy displayed lower efficacy and more non-healing wounds, as observed in those with wounds in both legs (cases 2 and 4) and in case 3, who had the worst efficacy scores. Incidentally, both patients died from myocardial infarction within 1 year after therapy, indicating that patients with low cardiac function, severe heel ulceration, and limb ischemia have a greater risk of mortality.^53^ Such patients may not be good candidates for cell therapy. Moreover, as some comorbid conditions are known to affect PAD outcomes,^54,55^ the relationship between patient background, EPC potential, and EPC efficacy in cell therapy should be explored further.

We investigated the relationship between the efficacy score and time to intervention for PTA. In patients with a greater efficacy score for MNC-QQ therapy, the time to PTA for restenosis after MNC-QQ therapy was much longer (12 months) than that in cases with lower efficacy scores (2-4 months). Stenotic lesions in blood vessels below the knee in patients undergoing dialysis often impede blood flow, and restenosis of the popliteal artery has been reported to occur in greater than 70% of patients after 3 months of PTA.^9^ Although MNC-QQ cells are only transplanted in the plantar region of the foot and increase the patency period of the lower limb artery, preventing restenosis may be difficult. Thus, MNC-QQ therapy may be more effective if the cells are transplanted in the lower limb and not just in the foot.

The limited sample size prevented us from evaluating the correlation between the efficacy score of cell therapy and number of MNC-QQ cells with specific CD markers that we studied previously.^22^ However, we plan to identify CD markers that can be used as reliable predictive biomarkers of efficacious MNC-QQ cell therapy, such as either the percentage positivity or total cell number of CD34 and CD206 cell markers of MNC-QQ potency in the release test.

Angiogenesis in patients with diabetes is highly inflammatory and compromised.^56^ We previously reported that PBMNC characteristics during autologous cell therapy in patients with diabetes are proinflammatory.^23^ In contrast, post-QQ culture treatment resulted in increased CD206^+^ cells, CD34^+^/CD133^+^ cells, angiogenic T cells, and regulatory T cells in all cases, along with lower levels of CCR2^+^, CD56^+^, and CD19^+^ cells (Supplementary Fig. 2). Under QQc conditions, PBMNCs may selectively increase the stem cell population of EPCs, anti-inflammatory and proangiogenic monocytes, and T lymphocytes while eliminating both proinflammatory and anti-regenerative cells. As it was impossible to track neovascularization after injecting the cells, we could not determine the mechanism of MNC-QQ cell-mediated improvement in vascular perfusion and wound healing. However, the results from our preclinical murine study showed that MNC-QQ cell therapy directly accelerated wound closure, maturation, and vascularization in both diabetic and euglycemic wounds by upregulating matrix metalloproteinase-9 and transforming growth factor-beta gene expression.^23^ Finally, although mild and transient AEs were frequent, severe AEs or development of emboli, malignant tumors, or angina pectoris did not occur during our trial.

Despite many innovations in cell-based therapy of diabetic ulcers, successful translation of this research from preclinical to clinical use in humans is limited, as several cell-based therapeutics, including CureXcell, by MacroCure Ltd., showed a loss of efficacy in phase III clinical trials.^57^ In summary, this prospective clinical trial is the first demonstration that autologous MNC-QQ cell transplantation may be an alternative therapy for treating chronic non-healing extremity wounds in patients with peripheral vascular disease or chronic renal failure requiring hemodialysis. This approach has several advantages. First, the ability to harvest sufficient numbers of functional MNCs from only 200 mL of PB makes the procedure minimally invasive and more convenient than other stem cell therapies. Second, the MNC-QQ process enables restoration of the vasculogenic function of EPCs, reducing the short-term need for further PTA and helping wound repair without any serious AEs in patients with diabetes.

Nevertheless, the heterogeneity of the population of patients with diabetes who presented with foot ulceration was a limitation of this study. Additionally, our study was limited by the small sample size, absence of a placebo-controlled group, and specific location of the ulcers. However, we included more patients compared to a recent clinical study involving transplantation of G-CSF-mobilized PB CD34^+^ cells in 6 patients with CLTI undergoing hemodialysis.^58^ Moreover, in this safety and feasibility study, we examined both the functional and clinical outcomes of the transplanted cells and compared them with the outcomes from the previous study. The findings indicate that our MNC-QQ cell therapy was effective and safe, even for patients with diabetes who had severe CLTI and large wounds. In addition, the 1-year cardiovascular event-free survival rate (78%) was higher in our study than in the earlier report.^58^ Age, blood fibrinogen, arterial occlusion above the knee, TcPO_2_, and transplanted CD34^+^ cell counts have been reported as predictors of the response to CD34^+^ cell therapy in patients with no-option CLTI.^59^

## Conclusion

This is the first human clinical trial of autologous PBMNC-QQ transplantation. We demonstrated that the procedure is safe and minimally invasive and is an effective therapy for patients with chronic non-healing ischemic extremity wounds. Larger clinical studies that include proper control groups are needed to establish the safety and efficacy of the procedure.

## Supplementary Material

szab018_suppl_Supplementary_Figure_S1

szab018_suppl_Supplementary_Figure_S2

szab018_suppl_Supplementary_Tables

szab018_suppl_Supplementary_Checklist

## Data Availability

The datasets used and/or analyzed during the present study are available from the corresponding author upon request.
