# Supplementary figures and images for: Phase I/IIa Feasibility Trial of Autologous Quality- and Quantity-Cultured Peripheral Blood Mononuclear Cell Therapy for Non-Healing Extremity Ulcers
Source: Stem Cells Transl Med. 2022 Feb 26;11(2):146–58. doi: 10.1093/stcltm/szab018 (PMC8929435; doi:10.1093/stcltm/szab018)

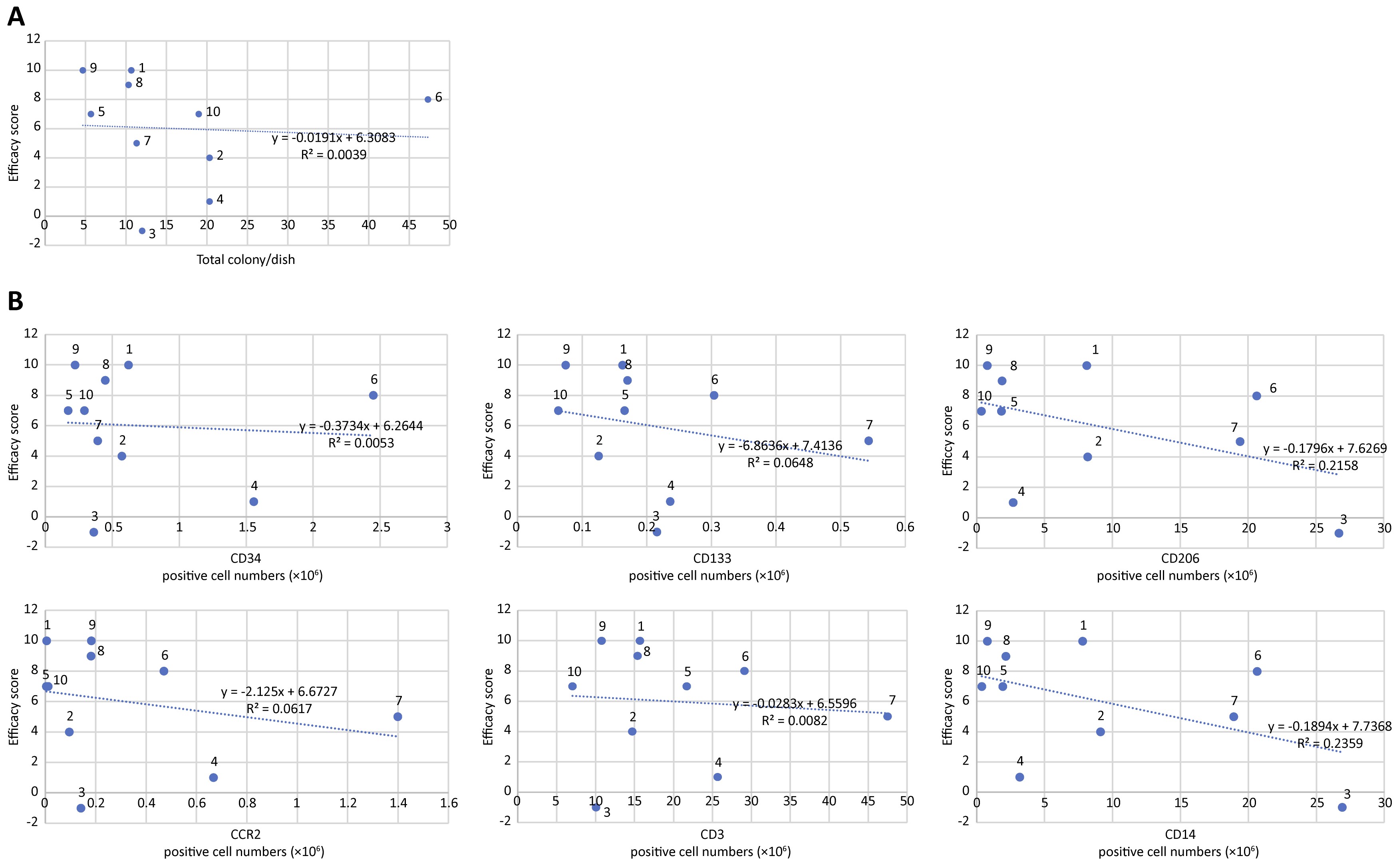

Supplement: szab018_suppl_Supplementary_Figure_S1 [file szab018_suppl_supplementary_figure_s1.jpeg]

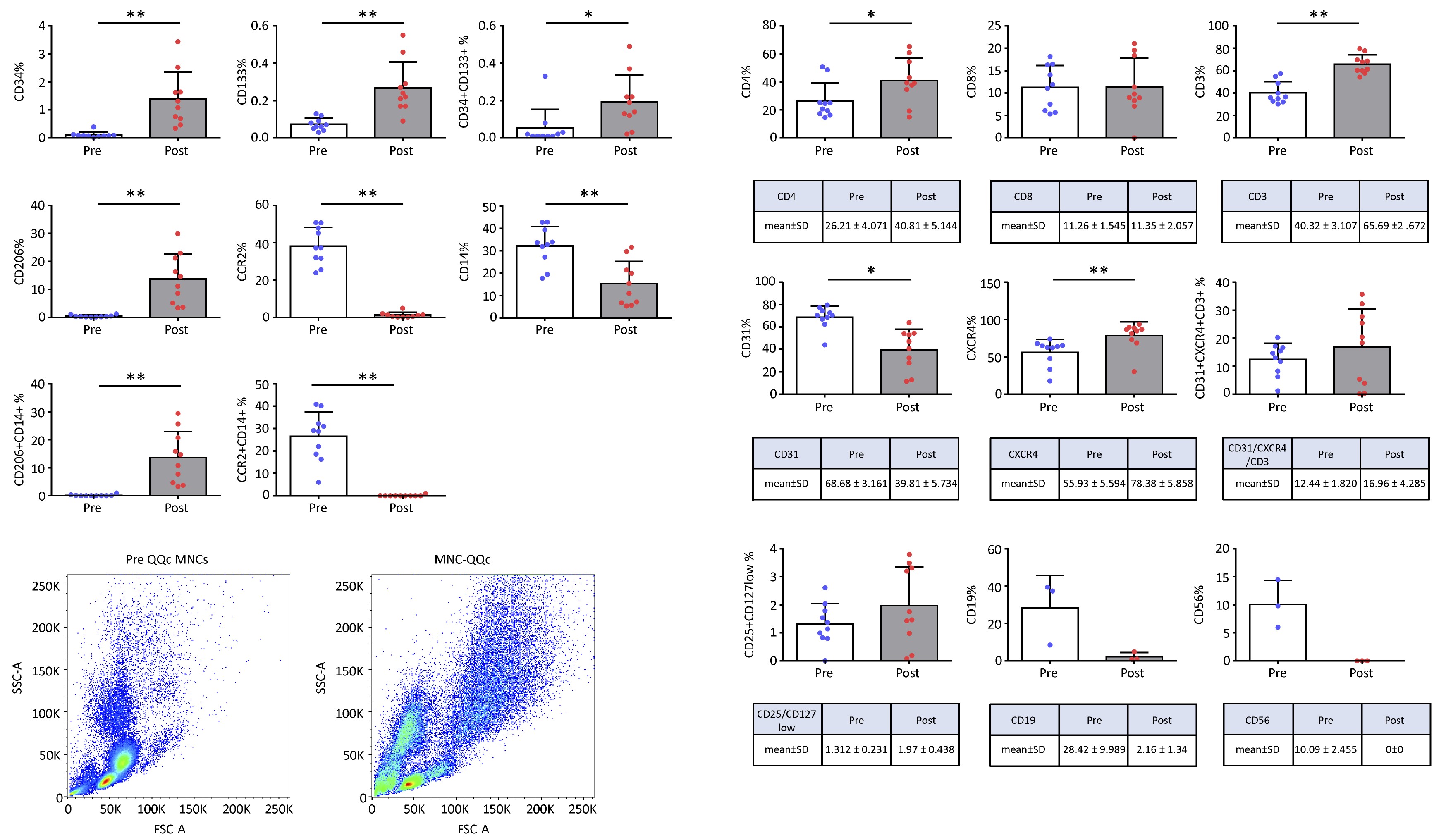

Supplement: szab018_suppl_Supplementary_Figure_S2 [file szab018_suppl_supplementary_figure_s2.jpeg]
