# Supplementary material for: Phase I/IIa Feasibility Trial of Autologous Quality- and Quantity-Cultured Peripheral Blood Mononuclear Cell Therapy for Non-Healing Extremity Ulcers
Source: Stem Cells Transl Med. 2022 Feb 26;11(2):146–58. doi: 10.1093/stcltm/szab018 (PMC8929435; doi:10.1093/stcltm/szab018)
Supplement: szab018_suppl_Supplementary_Tables [file szab018_suppl_supplementary_tables.docx]

**Supplementary Tables**

**Supplementary Table 1.** Calculation of efficacy scores

| **Parameter** |  | **Score value** |
| --- | --- | --- |
|  | **Wound closure after 12 weeks score according to the difference from baseline** | |
| 76%–100% wound closure |  | **+2** |
| 36%–75% wound closure |  | **+1** |
| 0%–35% wound closure |  | **0** |
| Wound enlargement |  | **−1** |
| Minor amputation |  | **−2** |
|  | **SPP score 12 weeks after treatment** | |
| >40 mmHg |  | **+2** |
| 30 mmHg < SPP < SPP40 mmHg |  | **+1** |
| <30 mmHg |  | **0** |
| No change |  | **−1** |
| Worsening |  | **−2** |
|  | **Wong–Baker FACES Pain Rating Scale score 12 weeks after treatment** | |
| Improvement by ≧2 steps |  | **+2** |
| Improvement by 1 step |  | **+1** |
| No change |  | **0** |
| Worsening by 1 step |  | **−1** |
| Worsening by ≧2 steps |  | **−2** |
|  | **Recurrence on the treated limb** | |
| No recurrence more than a year |  | **+2** |
| No recurrence within a year |  | **+1** |
| Recurrence but healed |  | **0** |
| Recurrence but non-healing |  | **−1** |
| Recurrence with amputation |  | **−2** |
|  | **Amputation-free survival at 1 year after therapy** | |
| Survival |  | **+1** |
| Death |  | **−1** |
|  | **Recurrence of ischemia** | |
| None |  | **+1** |
| Treated with intervention |  | **−1** |

Efficacy score = (% wound closure + SPP on treated foot + Wong–Baker FACES Pain Scale on the treated leg) after 12 weeks of therapy + recurrence of wound + recurrence of ischemia + amputation free survival at one year after therapy. Range: −2 to +2 for the first four categories; −1 to +1 for the last two categories. SPP, skin perfusion pressure

**Supplementary Table 2.** Product outcome and release test results of peripheral blood MNC-QQ cells in all cases

| Cases | **Pre QQ** | | **MNC-QQ** | | | **Safety test** | | | **Post QQ FACS data** | | | | | |
| --- | --- | --- | --- | --- | --- | --- | --- | --- | --- | --- | --- | --- | --- | --- |
|  | **Total cell number (×10^6^)** | **Live cell (%)** | **Post QQ culture cell number (×10^6^)** | **Live cell post QQ (%)** | **Fold Increase** | **Endotoxin**  **（<0.25 EU/mL）** | **Mycoplasma** | **Bacterial culture test** | **CD34** | **CD206** | **CCR2** | **CD133** | **CD3** | **CD14** |
| Case 1 | 150.0 | 76.9 | 29 | 60.5 | 0.196 | <0.001 | Negative | All Negative | 2.14 | 28 | 0.02 | 0.56 | 54.15 | 26.9 |
| Case 2 | 232.0 | 100.0 | 33 | 86.8 | 0.143 | <0.001 | Negative | All Negative | 1.73 | 24.8 | 0.29 | 0.38 | 44.51 | 27.58 |
| Case 3 | 180.0 | 93.3 | 49 | 82.9 | 0.269 | <0.001 | Negative | All Negative | 0.74 | 54.5 | 0.29 | 0.44 | 20.52 | 54.9 |
| Case 4 | 243.0 | 98.4 | 40 | 87 | 0.167 | <0.001 | Negative | All Negative | 3.89 | 6.7 | 1.67 | 0.59 | 64.19 | 7.89 |
| Case 5 | 212.0 | 99.5 | 29 | 76.3 | 0.137 | <0.001 | Negative | All Negative | 0.59 | 6.27 | 0.04 | 0.57 | 74.88 | 6.6 |
| Case 6 | 280.0 | 71.7 | 62 | 83.8 | 0.231 | <0.001 | Negative | All Negative | 3.95 | 33.28 | 0.76 | 0.49 | 46.94 | 33.28 |
| Case 7 | 256.0 | 99.0 | 76.5 | 84.5 | 0.299 | <0.001 | Negative | All Negative | 0.51 | 25.39 | 1.83 | 0.71 | 62.09 | 24.72 |
| Case 8 | 217.5 | 100.0 | 20.5 | 78.8 | 0.094 | <0.001 | Negative | All Negative | 2.18 | 9.17 | 0.89 | 0.83 | 75.12 | 10.44 |
| Case 9 | 94.3 | 99.5 | 14.6 | 84.5 | 0.159 | <0.001 | Negative | All Negative | 1.52 | 5.35 | 1.26 | 0.51 | 73.69 | 5.3 |
| Case 10 | 198.5 | 88.8 | 9.3 | 74.4 | 0.05 | <0.001 | Negative | All Negative | 3.14 | 3.9 | 0.05 | 0.68 | 75.56 | 4.05 |

* The pre-QQ denotes freshly isolated MNCs.

Fold increase depicts the decrease in MNC numbers after QQ culture.

Safety tests included tests for endotoxin, mycoplasma, and bacterial culture.

Post-QQ MNC cells were tested for cell surface markers CD34, CD206, CCR2, CD133, CD3, and CD14. The numbers are in percent positivity.

**Supplementary Table 3.** Efficacy scores of all the patients after 12 weeks of PBMNC-QQ therapy

|  | Wound healing | vascular perfusion | Pain | Recurrence | 1yrAFS | Restenosis | Total |
| --- | --- | --- | --- | --- | --- | --- | --- |
| **Case 1** | 2 | 2 | 2 | 2 | 1 | 1 | 10 |
| **Case2** | 1 | 2 | 2 | 1 | −1 | −1 | 4 |
| **Case 3** | −2 | 2 | 0 | 1 | −1 | −1 | −1 |
| **Case 4** | −1 | 2 | 1 | 1 | −1 | −1 | 1 |
| **Case 5** | 2 | 2 | 1 | 0 | 1 | 1 | 7 |
| **Case 6** | 2 | 2 | 2 | 0 | 1 | 1 | 8 |
| **Case 7** | 2 | 2 | 1 | 0 | 1 | −1 | 5 |
| **Case 8** | 2 | 2 | 1 | 2 | 1 | 1 | 9 |
| **Case 9** | 2 | 2 | 2 | 2 | 1 | 1 | 10 |
| **Case 10** | 2 | 2 | 2 | 1 | 1 | −1 | 7 |
